# *IL1* genes polymorphism and the risk of renal cell carcinoma in Chinese Han population

**DOI:** 10.18632/oncotarget.18715

**Published:** 2017-06-28

**Authors:** Fei Wang, Yingai Zhang, Shunlan Wang, Yadong Zhang, Dinglan Wu, Chong Zhang, Yuanhui Gao, Xi Liu, Weifu Wang, Shufang Zhang

**Affiliations:** ^1^ Department of Urology, People's Hospital of Hainan Province, Haikou, Hainan, 570311, P.R. China; ^2^ Central Laboratory, Haikou People's Hospital, Central South University Xiangya School of Medicine Affiliated Haikou Hospital, Haikou, Hainan, 570208, P.R. China; ^3^ Department of Urology, First Affiliated Hospital, Sun Yat-Sen University, Guangzhou, Guangdong, 510080, P.R. China; ^4^ Central Laboratory, Shenzhen Hospital, Southern Medical University, Shenzhen, Guangdong, 518110, P.R. China; ^5^ Department of Urology, Haikou People's Hospital, Central South University Xiangya School of Medicine Affiliated Haikou Hospital, Haikou, Hainan, 570208, P.R. China

**Keywords:** renal cell carcinoma (RCC), case-control studies, IL1, single-nucleotide polymorphism (SNP)

## Abstract

Renal cell carcinoma (RCC) is considered a cytokine-responsive tumor. However, with the lack of diagnostic screening biomarkers, early diagnosis of RCC is challenging. Our study was investigated the association of IL1 gene polymorphisms and RCC risk. We conducted a case-control study of 291 RCC cases and 463 controls to evaluation the IL1RN of single nucleotide polymorphisms (SNPs) on RCC risk. We selection of 16 SNPs in IL1RN, IL1A, IL1B genes were analyzed. Using the chi-squared (χ^2^) test and genetic model analysis, we found an association with RCC risk for five SNPs [rs3783550 (IL1A), rs3783546 (IL1A), rs1609682 (IL1A), rs3783521 (IL1A), and rs1143623 (IL1B)] and increased the risk of RCC. Stratified analyses show that smoking, not drinking and age>55 populations relative to nonsmoking, drinking and age<55 more susceptible. Our study suggested that IL1B and IL1A may involve in the development of RCC in Chinese Han population.

## INTRODUCTION

Kidney cancer accounting for 2% of all malignancies, it has become one of the most important health problems worldwide, cases are increasing year by year [1]. Renal cell carcinoma (RCC) is the major cancer type in kidney, and accounts for about 3% of all human malignancies, with a male-to-female ratio of approximately 2:1 [1, 2]. RCC risk factors included smoking tobacco [3], obesity [4, 5], hypertension [6], and a history of chronic kidney disease [7]. Besides the environmental risk factors, inherited risk has been considered crucial risk factors of RCC. A recent study has demonstrated that genetic factors contribute greatly to the occurrence of RCC. [8, 9]. Genome-wide association studies (GWAS) have shown EPAS1, HIF-2α, CCND1, ITPR2 were significantly associated with RCC susceptibility in western populations [10–13]. However, these GWAS identified RCC risk SNPs identified in western populations was rarely replicated in Chinese populations. The potential role of RCC risk in immune response has been demonstrated [14, 15]. RCC tissue is often infiltrated by lymphocytes, macrophages and dendritic cells, reflecting the immunogenicity of RCC [16–18]. The present pathogenic mechanism of RCC, because of these two inflammatory conditions, is characterized by the presence of a wide range of proinflammatory cytokines. It has also been shown a variety of cytokines such as tumor necrosis factor alpha (TNF- alpha), interleukin -6 (IL-6), type 1 interferons (IFNs) and interferon gamma *in vitro* [19–22]. The effects of cytokines observed in these studies were either growth promoting or antiproliferative [21]. In addition, polymorphisms in the immune response may explain human diversity because they lead to differences in the ability of individuals to produce cytokines, resulting in a wide variety of biological consequences. Although many studies investigating the frequency of cytokine gene polymorphisms appear in chronic kidney disease patients, there is still a lack of IL1 regarding how renal cancer can be related. Therefore, the purpose of this study was to investigate the IL-1 cytokine gene polymorphism and cytokine receptor gene polymorphism in RCC patients.

## RESULTS

Demographic and clinical features of the RCC and the control group are shown in Table 1. The cases were well matched with the controls in terms of age (P = 0.273) and gender (P = 0.017), smoking status (P = 0.193). However, drinking status exist significant difference between two groups (P< 0.05). In Table 2, we listed the primers in the study. All SNPs were in Hardy–Weinberg equilibrium among control subjects, in addition to rs928940. The distributions of the allele frequencies for all selected polymorphisms are presented in Table 3. Our results indicated that polymorphism rs3783550, rs3783546, rs1609682 and rs3783521 in IL1A gene are associated with susceptibility to RCC. We hypothesized that harboring minor allele per SNP is a risk factor, compared with owning wild-type alleles. The results of various genetic models are shown in Table 4. Five SNPs were significantly associated with RCC risk in Chinese han population, and were used to establish the genetic risk models. Our results showed that the rs3783550 T allele carriers (dominant, Log-additive model) was associated with an increased risk of RCC (dominant, OR=1.39, 95%CI = 1.03-1.88, P= 0.031; Log-additive, OR=1.32, 95%CI = 1.05-1.64, P= 0.015). The genotype “C/C” and “G/C-C/C” of rs3783546 in IL1A gene was associated with of RCC risk in Codominant(OR = 1.77, 95% CI=1.09-2.87, p = 0.04), dominant model (OR=1.41, 95%CI = 1.04-1.91, P= 0.025). For rs1609682, carriers “T” allel individual, increased RCC risk (OR=1.32, 95%CI = 1.06-1.65, P= 0.013). The genotype “G/G” and “G/A-G/G” of rs3783521 was associated with an increased risk of RCC in Codominant, dominant, and Log-additive model (OR = 1.33, 95% CI= 1.06-1.66, p = 0.012). Meanwhile, we adjustment for age, sex, we found rs1143623(G) were associated with RCC risk in recessive model(OR = 1.54, 95% CI= 1.02-2.32, p = 0.04).

**Table 1:** Characteristics of RCC patients and control participants

Variable	Case	Control	P
Total	291	463	
Age(Mean±SD)	56.88±11.66	50.65±11.79	0.273
BMI(Mean±SD)	23.96±2.90	23.79±3.77	0.098
Gender			
Female	99	198	0.017
Male	192	265	
Smoking status			
Smoking	120	169	0.193
Nosmoking	171	294	
Drinking status			
Drinking	53	174	<0.050
Nodrinking	238	289	

**Table 2:** PCR primers

SNP	1st-PCR primer sequences	2st-PCR primer sequences	UEP sequences
rs3783550	ACGTTGGATGTGAAGGCCAAATGCTAAGGG	ACGTTGGATGCTCAGGCATCTCCTATGAAG	cGAATTCTGTTAGAGAACAAGATG
rs3783546	ACGTTGGATGACACTGCTGTTGGCACTATG	ACGTTGGATGGTAGGGCAGTAGCTTCATTC	ccTTATTCACTGAGAGCCTT
rs2856838	ACGTTGGATGCTGGTGTCAGAGAAGACAAC	ACGTTGGATGGTTAGTTATGCCATCCTGAG	CCAGGTGTCTGTCTCCTAA
rs1609682	ACGTTGGATGCATGGGACTGCTATTCTTAC	ACGTTGGATGACCTCTAGTGAGGGTAAAAC	gAGTGAGGGTAAAACAAAAGTATT
rs3783521	ACGTTGGATGCCAACTGGCTACATTTCTGC	ACGTTGGATGTCAGGAGGAGAGGGTTAATC	ccTGTTGCCTAAAGAGGAA
rs2853550	ACGTTGGATGCGAAGACTATCCTCCTCACC	ACGTTGGATGTGCAGTGCTTCAGCTGATCC	CAGCTGATCCTGTTCCA
rs1143643	ACGTTGGATGCCTCAGCATTTGGCACTAAG	ACGTTGGATGACTCCTGAGTTGTAACTGGG	GGGCCCCCAACTTTC
rs3136558	ACGTTGGATGAAGGGCTTGAAAGAATCCCG	ACGTTGGATGGATTCATCCACCTCGGCTTC	aaccCGCCTGGCCCAGAGAGGGATGA
rs1143630	ACGTTGGATGTCTTGAGTCTGCCTCTAACC	ACGTTGGATGAGATTATCCCTCTCTGAAGC	AGCTCAAGGAGGTTAAG
rs1143627	ACGTTGGATGTCTCAGCCTCCTACTTCTGC	ACGTTGGATGTTGTGCCTCGAAGAGGTTTG	gtTCCCTCGCTGTTTTTAT
rs16944	ACGTTGGATGCTGTCTGTATTGAGGGTGTG	ACGTTGGATGAGAGGCTCCTGCAATTGACA	AATTGACAGAGAGCTCC
rs1143623	ACGTTGGATGACCTATTTCCCTCGTGTCTC	ACGTTGGATGATGTGCCAGGTATCGTGCTC	tttaGTGCTCGCTCTGCATTAT
rs17042888	ACGTTGGATGTGGAGTTGGAGTCTTGTTGG	ACGTTGGATGCTACTTGCTCAGCACCATAC	agcGGTGTTGAAATCCCAAAA
rs928940	ACGTTGGATGACATGGTTCCATCTCTATCC	ACGTTGGATGAGAAGAGAAAAGTTGACGGG	aTGACGGGGTGCATACTC
rs3181052	ACGTTGGATGCTTTATGTTTGTCTGGGCCG	ACGTTGGATGACAGTCCCCATATCTGGAAG	cttaACTCATACACCCACAGAGCC
rs452204	ACGTTGGATGAAAAGAGCCTCAACATGCAG	ACGTTGGATGTAGACTTAGCCACGTGACTG	gcccATAGGATGATGCAAGCAGAAGT

**Table 3:** Basic information of candidate SNPs in this study

SNP	Chr	Gene	allel	MAF(case)	MAF(control)	HWE	OR	95%CI		P
rs3783550	2q13	IL1A	T/G	0.38	0.33	0.399	1.28	1.04	1.58	0.018*
rs3783546	2q13	IL1A	C/G	0.38	0.33	0.459	1.30	1.05	1.60	0.014*
rs2856838	2q13	IL1A	A/G	0.27	0.25	0.320	1.12	0.89	1.40	0.338
rs1609682	2q13	IL1A	T/G	0.38	0.33	0.398	1.29	1.05	1.59	0.016*
rs3783521	2q13	IL1A	G/A	0.38	0.33	0.399	1.29	1.05	1.59	0.015*
rs2853550	2q13	IL1B	A/G	0.11	0.09	0.067	1.29	0.92	1.79	0.137
rs1143643	2q13	IL1B	C/T	0.48	0.47	0.191	1.01	0.83	1.23	0.926
rs3136558	2q13	IL1B	G/A	0.41	0.37	0.617	1.21	0.99	1.48	0.066
rs1143630	2q13	IL1B	T/G	0.18	0.16	1.000	1.16	0.89	1.51	0.262
rs1143627	2q13	IL1B	G/A	0.49	0.48	0.160	1.05	0.86	1.28	0.618
rs16944	2q13	IL1B	A/G	0.49	0.48	0.162	1.06	0.87	1.29	0.556
rs1143623	2q13	IL1B	G/C	0.41	0.40	0.286	1.04	0.85	1.28	0.675
rs17042888	2q13	IL1RN	A/G	0.27	0.26	0.470	1.05	0.84	1.31	0.697
rs928940	2q13	IL1RN	T/G	0.41	0.39	0.000	1.07	0.87	1.31	0.510
rs3181052	2q13	IL1RN	G/A	0.43	0.41	0.248	1.09	0.89	1.34	0.384
rs452204	2q13	IL1RN	G/A	0.36	0.35	1.000	1.06	0.86	1.30	0.594

*p value ≤ 0.05 indicates statistical significance; *A/B* stands for minor/major alleles on the control sample frequencies. *HWE*: Hardy–Weinberg equilibrium*; MAF*: minor allele frequency; OR: odds ratio; SNP: single-nucleotide polymorphisms.

**Table 4:** Association between IL1 polymorphisms genotypes and RCC risk under different genotypic models

	Model	Genotype	control	case	OR (95% CI)	P-value
rs3783550	Codominant	G/G	206 (44.5%)	106 (36.5%)	1	
		G/T	212 (45.8%)	144 (49.7%)	1.32 (0.96-1.81)	0.053
		T/T	45 (9.7%)	40 (13.8%)	1.73 (1.06-2.81)	
	Dominant	G/G	206 (44.5%)	106 (36.5%)	1	0.031*
		G/T-T/T	257 (55.5%)	184 (63.5%)	1.39 (1.03-1.88)	
	Recessive	G/G-G/T	418 (90.3%)	250 (86.2%)	1	0.089
		T/T	45 (9.7%)	40 (13.8%)	1.49 (0.94-2.34)	
	Log-additive	---	---	---	1.32 (1.05-1.64)	0.015*
rs3783546	Codominant	G/G	206 (44.7%)	106 (36.4%)	1	0.040*
		G/C	210 (45.5%)	144 (49.5%)	1.33 (0.97-1.83)	
		C/C	45 (9.8%)	41 (14.1%)	1.77 (1.09-2.87)	
	Dominant	G/G	206 (44.7%)	106 (36.4%)	1	0.025*
		G/C-C/C	255 (55.3%)	185 (63.6%)	1.41 (1.04-1.91)	
	Recessive	G/G-G/C	416 (90.2%)	250 (85.9%)	1	0.072
		C/C	45 (9.8%)	41 (14.1%)	1.52 (0.97-2.38)	
	Log-additive	---	---	---	1.33 (1.07-1.66)	0.011*
rs1609682	CoDominant	G/G	205 (44.4%)	106 (36.4%)	1	0.046*
		G/T	212 (45.9%)	144 (49.5%)	1.31 (0.96-1.80)	
		T/T	45 (9.7%)	41 (14.1%)	1.76 (1.09-2.86)	
	Dominant	G/G	205 (44.4%)	106 (36.4%)	1	0.031*
		G/T-T/T	257 (55.6%)	185 (63.6%)	1.39 (1.03-1.88)	
	Recessive	G/G-G/T	417 (90.3%)	250 (85.9%)	1	0.070
		T/T	45 (9.7%)	41 (14.1%)	1.52 (0.97-2.39)	
	Log-additive	---	---	---	1.32 (1.06-1.65)	0.013*
rs3783521	CoDominant	A/A	206 (44.5%)	106 (36.4%)	1	0.043*
		G/A	212 (45.8%)	144 (49.5%)	1.32 (0.96-1.81)	
		G/G	45 (9.7%)	41 (14.1%)	1.77 (1.09-2.87)	
	Dominant	A/A	206 (44.5%)	106 (36.4%)	1	0.028*
		G/A-G/G	257 (55.5%)	185 (63.6%)	1.40 (1.04-1.89)	
	Recessive	A/A-G/A	418 (90.3%)	250 (85.9%)	1	0.069
		G/G	45 (9.7%)	41 (14.1%)	1.52 (0.97-2.39)	
	Log-additive	---	---	---	1.33 (1.06-1.66)	0.012*
rs1143623	CoDominant	C/C	159 (34.6%)	110 (37.9%)	1	0.065
		C/G	233 (50.6%)	124 (42.8%)	0.82 (0.59-1.16)	
		G/G	68 (14.8%)	56 (19.3%)	1.38 (0.88-2.17)	
	Dominant	C/C	159 (34.6%)	110 (37.9%)	1	0.720
		C/G-G/G	301 (65.4%)	180 (62.1%)	0.94 (0.69-1.30)	
	Recessive	C/C-C/G	392 (85.2%)	234 (80.7%)	1	0.040*
		G/G	68 (14.8%)	56 (19.3%)	1.54 (1.02-2.32)	
	Log-additive	---	---	---	1.10 (0.88-1.37)	0.400

**Figure 1:**
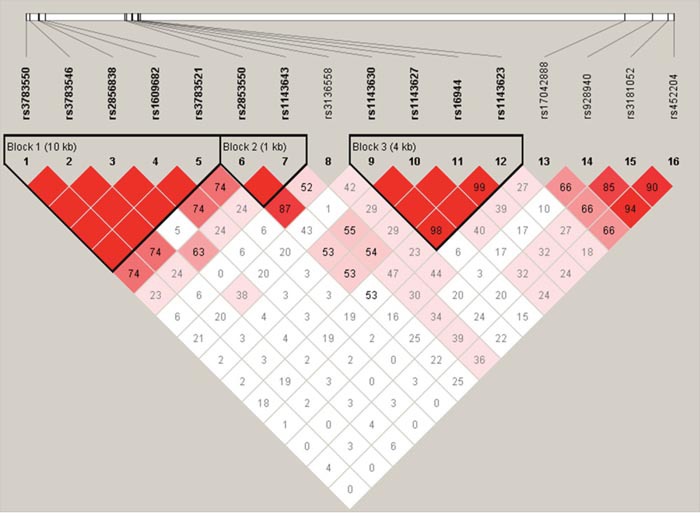
Haplotype block map for the IL1 SNPs genotyped in this study

The LD and haplotype analysis were investigated. We found one block in studied IL1A SNPs (Figure 1). In Table 5 we listed the association between IL1A haplotype and the risk of RCC. Haplotype estimation analysis showed that the haplotype of rs3783550T/ rs3783546C/ rs2856838G/ rs1609682T/rs3783521G(TCGTG) It could be a potential risk factor for RCC (OR = 1.68, 95% CI= 1.19 - 2.37, p = 0.0032).

**Table 5:** Haplotype frequencies and their associations with RCC risk

Block	rs3783550	rs3783546	rs2856838	rs1609682	rs3783521	Freq	OR (95% CI)	P-value
1	G	G	G	G	A	0.6499	1	---
2	T	C	A	T	G	0.2555	1.20 (0.93 - 1.54)	0.160
3	T	C	G	T	G	0.0946	1.68 (1.19 - 2.37)	0.003*

In addition we also carried out smoking, drinking, age stratification analysis, we found in smoking population there have six SNPs rs3783550 (P=0.029), rs3783546 (P =0.019), rs1609682 (P=0.023), rs3783521 (P=0.021), rs1143630 (P=0.045), rs3136558 (P=0.020) polymorphism were associated with RCC, however, in nosmoking population we did not found any SNPs with RCC. Meanwhile, in nodrinking there was also a significant relationship between the risk of RCC of six SNPs except rs1143630 and rs3136558. For age > 55, five SNPs polymorphism (rs3783550 (P=0.003), rs3783546 (P =0.003), rs2856838 (P=0.020), rs1609682 (P=0.003), rs3783521 (P=0.003) were associated with RCC risk (Table 6).

**Table 6:** The association between SNPs and smoking, drinking status, age analysis of RCC patients

SNP	Allel	Smoking status				Drinking status				Age			
		Smoking		Nosmoking		Drinking		Nodrinking		>55		≤55	
		OR(95%CI)	*P*	OR(95%CI)	*P*	OR(95%CI)	*P*	OR(95%CI)	*P*	OR(95%CI)	*P*	OR(95%CI)	*P*
rs3783550	T/G	1.37(1.03-1.81)	0.029	1.20(0.85-1.68)	0.308	0.95(0.60-1.50)	0.823	1.43(1.11-1.85)	0.006	1.43(1.11-1.85)	0.003	1.01(0.74-1.38)	0.959
rs3783546	C/G	1.39(1.05-1.84)	0.019	1.20(0.85-1.68)	0.308	0.95(0.60-1.51)	0.836	1.45(1.13-1.87)	0.004	1.45(1.13-1.87)	0.003	1.03(0.76-1.41)	0.831
rs2856838	A/G	1.18(0.87-1.61)	0.284	0.99(0.68-1.43)	0.941	0.93(0.57-1.53)	0.774	0.83(0.63-1.10)	0.198	0.83(0.63-1.10)	0.020	0.81(0.57-1.14)	0.227
rs1609682	T/G	1.38(1.04-1.82)	0.023	1.20(0.85-1.68)	0.308	0.95(0.60-1.50)	0.823	1.44(1.12-1.85)	0.005	1.44(1.12-1.85)	0.003	1.03(0.75-1.41)	0.845
rs3783521	G/A	1.39(1.05-1.83)	0.021	1.20(0.85-1.68)	0.308	0.95(0.60-1.50)	0.823	1.45(1.12-1.86)	0.004	1.45(1.12-1.86)	0.003	1.03(0.75-1.41)	0.845
rs2853550	A/G	1.43(0.93-2.21)	0.106	1.17(0.66-2.06)	0.591	0.80(0.36-1.80)	0.589	1.49(0.99-2.23)	0.052	1.49(0.99-2.23)	0.142	1.26(0.78-2.03)	0.340
rs1143643	C/T	1.10(0.84-1.43)	0.495	1.30(0.93-1.82)	0.121	0.95(0.62-1.48)	0.836	1.01(0.79-1.29)	0.939	1.01(0.79-1.29)	0.453	0.98(0.73-1.31)	0.869
rs3136558	G/A	1.38(1.05-1.82)	0.020	0.94(0.67-1.32)	0.708	1.11(0.71-1.74)	0.644	1.20(0.93-1.53)	0.157	1.20(0.93-1.53)	0.817	1.36(1.01-1.84)	0.044
rs1143630	T/G	1.43(1.01-2.04)	0.045	0.82(0.53-1.28)	0.381	0.79(0.43-1.46)	0.453	0.77(0.56-1.07)	0.114	0.77(0.56-1.07)	0.162	1.06(0.72-1.56)	0.764
rs1143627	G/A	1.03(0.78-1.34)	0.851	1.10(0.79-1.54)	0.568	0.95(0.61-1.47)	0.814	1.03(0.81-1.32)	0.803	1.03(0.81-1.32)	0.414	0.98(0.73-1.32)	0.915
rs16944	A/G	1.03(0.79-1.35)	0.803	1.11(0.79-1.54)	0.548	0.96(0.62-1.48)	0.847	1.04(0.82-1.33)	0.738	1.04(0.82-1.33)	0.291	0.97(0.72-1.30)	0.834
rs1143623	G/C	0.97(0.74-1.27)	0.801	1.16(0.83-1.64)	0.378	1.02(0.65-1.60)	0.933	1.01(0.79-1.29)	0.944	1.01(0.79-1.29)	0.592	1.01(0.75-1.36)	0.965
rs17042888	A/G	0.82(0.60-1.11)	0.194	1.34(0.92-1.95)	0.129	0.89(0.53-1.49)	0.663	1.02(0.77-1.34)	0.915	1.02(0.77-1.34)	0.974	1.00(0.72-1.40)	0.996
rs928940	T/G	0.89(0.68-1.18)	0.429	1.20(0.86-1.69)	0.287	0.95(0.61-1.49)	0.824	1.03(0.80-1.32)	0.831	1.03(0.80-1.32)	0.528	1.14(0.84-1.54)	0.406
rs3181052	G/A	1.00(0.77-1.31)	0.983	1.13(0.80-1.57)	0.491	0.89(0.57-1.39)	0.620	1.10(0.86-1.41)	0.451	1.10(0.86-1.41)	0.226	1.33(0.99-1.79)	0.061
rs452204	G/A	0.93(0.70-1.23)	0.605	1.22(0.86-1.72)	0.268	1.06(0.67-1.67)	0.811	1.02(0.79-1.32)	0.876	1.02(0.79-1.32)	0.398	1.25(0.92-1.70)	0.159

## DISCUSSION

In our case-control study of RCC, we elaborated the associations of 16 SNPs in *IL1A, IL1B and IL1RN* with the risk of RCC in Chinese han population. Our study revealed that IL1A and IL1B SNPs were associated with susceptibility to RCC, but there was no significant association between SNP and the risk of RCC in IL1RN.

We selected locus 2q13 in IL1A, IL1B, IL1RN three candidate genes were analyzed. The proteins encoded by these genes belong to interleukin -1 (IL-1). The three molecules in the IL-1 cluster, namely, IL-1α, IL-1β, and interleukin-1 receptor antagonist (IL-1Ra), are encoded by the IL1A, IL1B, and IL1RN genes, respectively [23–25]. IL-1 cytokines are a major inflammatory cytokine and have been implicated in the mediation of acute and chronic inflammatory diseases. It is a key mediator of inflammation, with pleiotropic effects on several cells and signaling pathways. IL-1 is a pleiotropic cytokine that participates in stimulating immune responses, such as inflammation, and regulates cellular growth, differentiation, and movement of cells. It is secreted by viruses, bacteria, and fungi that react to various antigens [26]. IL-1 cytokines are known to contribute to the development and progression of a number of renal diseases [27, 28], and polymorphisms of the IL-1 family cytokine genes have been studied for associations with IgAN susceptibility, progression, and clinical manifestations, such as hematuria and proteinuria. Several associations have been reported between IL-1 cluster gene polymorphisms and IgAN. However, IL-1cluster gene polymorphisms did not reported in RCC. The IL gene reported more focused on asthma, liver cancer, breast cancer, oral cancer, cervical cancer, leukemia and other research, at about rs3783550, rs3783546, rs1609682, rs3783521 polymorphisms were renal cell carcinoma and the related reports.

Smoking has been regarded as one of the risk factors of RCC. The risk of RCC caused by polymorphism is particularly significant in the smoking population, which may be caused by cigarette smoking induced immune suppression, so that renal cell carcinoma escape immune surveillance, thereby promoting the occurrence and development of renal cell carcinoma [29]. Studies have reported that between alcohol consumption and risk of renal cell carcinoma has a dose-dependent relationship, namely alcohol consumption to achieve a certain degree of risk exists only a small amount of alcohol, but can play the role of is beneficial to the body [30], this can be a very good explanation to our other hierarchical analysis results, the relative risk of drinkers for non-drinkers carrying the variant genotype and higher risk of RCC. These risk factors and genetic factors interact to affect the susceptibility of renal cancer. However, due to the limitations of data collection, the study of environmental factors are limited to smoking, drinking and other factors, so it is not able to completely analyze the interaction of gene environment. Therefore, it is necessary to carry out a complete analysis of the gene environment interaction, and to verify the results of the study.

Our results need to be validated in a larger sample and in other races with functional analysis to clarify the potential mechanisms underlying the links between SNPs of IL-1 and susceptibility to RCC.

## MATERIALS AND METHODS

### Study subjects

The case-control study included 291 histopathologically confirmed RCC patients and 463 controls. None of the patients had a family history of carcinoma. A total of 291 patients with RCC were prospectively recruited from People's Hospital of Hainan Province, Haikou people's Hospital Affiliated to Xiangya Medical School of Central South University, and First Affiliated Hospital of Sun Yat-sen University, and 463 healthy controls from the same region were enrolled in this study. Detailed characteristics of the RCC patients and controls are shown in Table 1. The control group had no history of other tumors and was unrelated to the genes in RCC patients. General examinations, laboratory tests, and medical histories confirmed their health. Everyone personally asked the trained interviewer to collect information about demographic data and related factors using a pre-test questionnaire (smoking, drinking, BMI et al). This study was approved by People's Hospital of Hainan Province, Haikou people's Hospital Affiliated to Xiangya Medical School of Central South University, and First Affiliated Hospital of Sun Yat-sen University, and all individuals involved in this study have signed informed consent. After informed consent was issued by all individuals, 5-ml samples of venous blood were collected for genomic DNA extraction.

### Selection of single nucleotide polymorphisms and genotyping

Candidate SNPs were selected according to the following restrictions: (1) The SNPs located at IL1; (2) each SNP had minor allele frequency (MAF) of > 5% in Chinese Han population; (3) each SNP had an r^2^ of > 0.80. Thus, for this study, we have selected the 16 SNPs described (Table 2). Whole blood were extracted using GoldMag-Mini Whole Blood Genomic DNA Purification Kit (GoldMag Co. Ltd. Xi’an City, China). We measured DNA concentration using NanoDrop 2000 spectrophotometer. Sequenom MassARRAY Assay Design 3.0 Software was used to design a Multiplexed SNP MassEXTEND assay [31]. Sequenom MassARRAY RS1000 was used for genotyping, and the related data were managed using Sequenom Typer 4.0 Software [31, 32].

Laboratory personnel were blinded to the genotyping results of all samples.

### Statistical analysis

All SNPs performed Hardy Weinberg balance (HWE) performed by comparing the observed and expected frequencies of genotypes using χ^2^ analysis [33]. OR and 95%CI were calculated using the logistic regression model. The association between SNPs and RCC risk were analyzed by logistic regression (Four genetic models dominant, recessive, and additive model). The haplotype frequencies were determined using the SHEsis program.

Finally, we used statistical packages (4.2 Edition) to perform linkage disequilibrium (LD), haplotype genetic association construction, and polymorphism at loci [34, 35]. All p values were two-sided, and p <0.05 was indicated statistical significance.
